# The combination of prostate MRI PI-RADS scoring system and a genomic classifier is associated with pelvic lymph node metastasis at the time of radical prostatectomy

**DOI:** 10.1259/bjr.20220663

**Published:** 2023-02-20

**Authors:** Tarik Benidir, Zaeem Lone, Jane K Nguyen, Ryan Ward, Martin Hofmann, Eric A Klein, Omar Y Mian, Christopher J Weight, Andrei S Purysko

**Affiliations:** 1 Glickman Urological and Kidney Institute, Cleveland Clinic, Cleveland, Ohio, USA; 2 Robert J. Tomisch Pathology and Laboratory Medicine Institute, Cleveland Clinic, Ohio, USA; 3 Imaging Institute, Cleveland Clinic, Cleveland, Ohio, USA

## Abstract

**Objective:**

Pelvic lymph node metastasis (PLNM) at the time of radical prostatectomy (RP) portends an unfavorable prognosis in prostate cancer patients. Conventional and advanced imaging remains limited in its ability to detect PLNM. We sought to evaluate the combination of a genomic classifier Decipher with Prostate Imaging Reporting and Data System (PI-RADS) scores in improving the detection of PLNM.

**Methods:**

A retrospective review was performed of patients whom underwent RP, Decipher analysis, and pre-operative prostate MRI. Categorical variables were compared using Pearson chi-squareχ2 tests. Quantitative variables were assessed with Wilcoxon rank-sum tests. Multivariable logistic regression was used to identify predictors of PLNM on final pathology.

**Results:**

In total, 202 patients were included in the analysis, 23 of whom (11%) had PLNM. Patients with PLNM had higher median Decipher scores (0.73) than those without PLNM (0.61; p = 0.003). Patients with PLNM were more likely to demonstrate PI-RADS scores ≥ 4 (96%) than those without PLNM (74%; p = 0.012). Logistic regression demonstrated an interaction between Decipher score with PI-RADS score ≥4 (OR = 20.41; 95% CI, 2.10–198.74; p = 0.009) The combination demonstrated an area under the curve (AUC) of 0.73 (95% CI, 0.63–0.82; p < 0.001) for predicting PLNM.

**Conclusion:**

The combination of elevated Decipher genomic score (≥ 0.6) and clinically significant PI-RADS score (≥ 4) is associated with PLNM at the time of RP in a modern high-risk cohort of patients with PCaprostate cancer.

**Advances in knowledge:**

Prostate MRI and genomic testing may help identify patients with adverse pathology.

## Introduction

The presence of pelvic lymph node metastasis (PLNM) at the time of radical prostatectomy (RP) portends an unfavorable prognosis and is associated with an elevated risk of biochemical recurrence of prostate cancer (PCa).^1^ The improved accuracy of PLNM identification thus remains an area of high interest for therapeutic decision-making.

Both CT and MRI relies on lymph node size to identify PLNM.^2,3^ As such, conventional imaging is of limited diagnostic accuracy for the detection of normal-sized PLNM. Efforts have been made to enhance MRI’s ability to detect PLNM,^4,5^ and the introduction of prostate-specific membrane antigen positron emission tomography (PSMA PET) has also shown promise in staging high-risk PCa.^6^ Despite improvements, considerable limitations remain, especially when considering the ongoing low sensitivity of PSMA in detecting normal-sized PLNM.^6–8^

The Prostate Imaging Reporting and Data System (PI-RADS) was developed to help clinicians detect clinically significant PCa (csPCa) on multiparametric MRI (mpMRI).^9^ Higher PI-RADS scores are associated with higher PCa grade groups (GGs), more advanced disease stages, and increased risk of biochemical recurrence.^10,11^ Additionally, although PI-RADS was not intended for lymph node evaluation, recent studies suggest a possible association between PI-RADS score and the risk of PLNM.^4,12^

Several gene expression assays have been prospectively validated to predict the presence of adverse pathology or early metastasis after RP.^13–16^ These assays in conjunction with MRI-targeted fusion biopsies have also been found to successfully predict the risk of adverse pathology.^17^ Decipher, one of these gene expression assays, is used to assess genes involved in cell cycle progression, cellular differentiation, adhesion and motility, the androgen pathway, and immune modulation.^18^ Previous research has shown that MRI-visible lesions have significantly higher Decipher risk scores than MRI-invisible lesions and that PI-RADS scores for MRI index lesions correlate with increasing Decipher risk scores.^18^

It remains unknown how the combination of genomic scores and pre-operative MRI findings aids in predicting PLNM. We hypothesized that the combination of Decipher risk scores in conjunction with PI-RADS scores would associate with PLNM status at the time of RP more accurately than either modality alone. The clinical implications of such a finding would help optimize counseling for these patients and offer a potential change in management approaches.

## Methods and materials

This was an Institutional Review Board approved study (21–1186). This was a single-center retrospective review of data collected prospectively between 2009 and 2020. Patients were included if they had undergone RP and pelvic lymph node dissection (PLND); pre-operative prostate MRI with image quality sufficient for confident radiographic assessment; and if they had undergone targeted genomic analysis of the highest grade lesion at RP. Patients were excluded if they had received preoperative neoadjuvant hormone treatment or were missing any above factors.

### Multiparametric MRI protocol and interpretation

All MRI scans were performed on a 3T scanner without an endorectal coil (acquisition parameters provided in Supplementary Table 1). MR images were re-reviewed by an abdominal radiologist (ASP) with >10 years of experience reading prostate MR images, who was blinded to clinicopathological outcomes of interest. PI-RADS v. 2.1 was used for lesions detected on MRI.^9^ The MRI index lesion was defined as the lesion with the highest PI-RADS score. When >2 had the same PI-RADS score, the index lesion was defined as the lesion demonstrating the greatest likelihood of extraprostatic extension (EPE) or the largest of the lesions if they had similar likelihoods of EPE. Lymph nodes were considered positive based on previously reported size criteria of ≥1.0 cm in short axis diameter for oval nodes and ≥0.8 cm for rounded nodes.^19^

Supplementary Table 1.

### PLND and histopathological analysis

All RP and PLND procedures were performed by experienced urologists. Either an open or minimally invasive robotic approach was used based on the decision of the overseeing surgeon. Resected prostate and lymph node specimens were inked, fixed in formalin, and serially sectioned in 3 mm wide intervals. Prostate slices were subdivided into four quadrants and individually labeled. Once fixed in formalin, the resected prostate and lymph node specimens were embedded in paraffin blocks for hematoxylin and eosin (H&E) staining. Each H&E-stained slide was then reviewed by a dedicated genitourinary pathologist, who identified the location, and extension of PCa, including any adverse pathological features, and determined the final International Society of Urological Pathology (ISUP) GG. In this study, ISUP-GG ≥2 cancers were considered clinically significant.

### Genomic analysis

Once the area of highest-grade disease at RP was mapped, a core biopsy of this area was sent to Decipher for genome analysis. Through tissue microarray analysis, Decipher evaluates the transcriptome of the biopsy specimen and also assesses a predefined set of 22 genes previously shown to be upregulated in patients at risk of adverse pathology.^15^ The Decipher score is a continuous variable ranging from 0 to 1 and categorically includes high-risk (>0.6), moderate-risk (0.45–0.6), and low-risk (<0.45) scores for adverse pathology after RP.

### Statistical analysis

Categorical variables were compared using the Pearson χ^2^ test. Quantitative variables were assessed with the Wilcoxon rank-sum test. Multivariate binary logistic regression with interaction analysis was used to identify predictors of PLNM. Using receiver operating characteristic (ROC) curve analysis, the areas under the curve (AUCs) for MRI’s lymph node evaluation, PI-RADS score, Decipher score, and combinations thereof were analyzed in terms of PLNM status. Predictive probability models were conducted for various Decipher risk scores (0.2–1.0). All tests were two-sided, with *p* values <0.05 considered significant. Statistical analysis was performed using IBM SPSS 28.0.

## Results

### Baseline characteristics

A total of 202 patients were included in the analysis. Of these, 23 patients (11%) had PLNM on final pathology (PLNM+) and 179 did not (PLNM–). PLNM+ patients had a significantly higher median Decipher score (0.73) than PLNM– patients (0.61; *p* = 0.003), as well as significantly higher pre-operative prostate-specific antigen (PSA) level (PLNM+: 8.2 ng ml^−1^; PLNM–: 6.2 ng ml^−1^; *p* = 0.004). PLNM+ patients were more likely to have ISUP-GG ≥3 disease (PLNM+: 87%; PLNM–: 51%; *p* = 0.006) and pathologic T3 disease (PLNM+: 92%; PLNM–: 70%; *p* = 0.003). PLNM+ patients were more likely to have disease categorized as PI-RADS score ≥4 on pre-operative MRI (PLNM+: 96%; PLNM–: 74%; *p* = 0.012). PLNM+ patients had a higher median number of nodes retrieved (12; interquartile range [IQR], 9–15) than PLNM– patients (8; IQR, 5–11; *p* = 0.002). Table 1 details pathologic differences between groups based on pelvic lymph node invasion status.

**Table 1.** Pre-operative patient characteristics

Characteristic	PLNM– (*n* = 179)	PLNM+ (*n* = 23)	*p*-value
Median length of follow-up, mo (IQR)	28 (21–86)	35 (23–48)	0.78
Age, yr (IQR)	64 (52–62)	66 (62–67)	0.15
Median preoperative PSA level, ng/mL (IQR)	6.2 (3.2–6.6)	8.2 (6.1–13.7)	**0.004**
Median Decipher score (IQR)	0.61 (0.29–0.54)	0.73 (0.68–0.87)	**0.003**
Decipher genomic risk class, n (%)	-	-	**0.005**
Low risk	55 (31)	2 (8)	
Intermediate risk	32 (18)	1 (4)	
High risk	92 (51)	20 (88)	
Pathologic T stage, n (%)	-	-	**0.003**
T2	53 (30)	2 (8)	
T3a	102 (57)	12 (52)	
T3b	24 (13)	9 (40)	
Pathologic ISUP-GG, n (%)	-	-	**0.006**
1	5 (3)	0 (0)	
2	83 (46)	3 (13)	
3	56 (31)	10 (43)	
4	14 (8)	2 (9)	
5	21 (12)	8 (35)	
EPE invasion, n (%)	125 (70)	21 (9)	**0.03**
Seminal vesicle invasion, n (%)	25 (14)	11 (48)	**< 0.001**
Median number of nodes retrieved (Q1–Q3)	8 (5-11)	12 (9–15)	**0.002**
Median number of positive nodes (Q1–Q3)	-	1 (1-2)	-
PI-RADS group, n (%)	-	-	**0.012**
1	10 (6)	0 (0)	
2	12 (7)	1 (4)	
3	22 (13)	0 (0)	
4	57 (34)	3 (13)	
5	78 (40)	19 (83)	

EPE = extraprostatic extension; IQR = interquartile range; ISUP-GG = International Society of Urological Pathology Grade Group; PI-RADS = Prostate Imaging Reporting and Data System; PSA = prostate-specific antigen.

### Diagnostic performance and predictors of PLNMon final pathology

**Figure 1.**
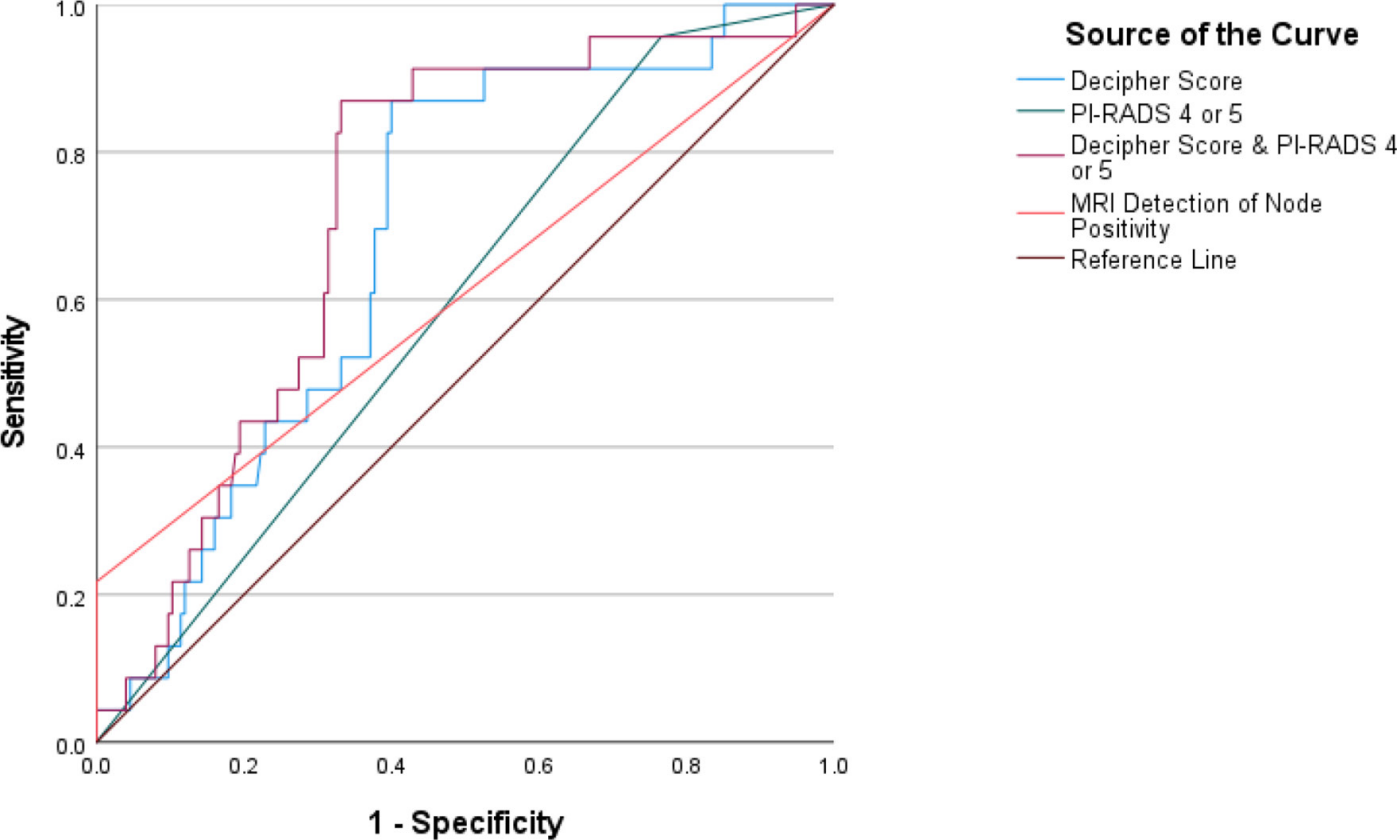
Receiver operating characteristic curve analysis. PI-RADS = Prostate Imaging Reporting and Data System.

On multivariate logistic regression (Table 2), the interaction between elevated Decipher score and PI-RADS score ≥4 (odds ratio [OR] = 20.41; 95% confidence interval [CI], 2.10–198.74; *p* = 0.009) was associated with PLNM, more so than other meaningful variables including pre-operative PSA. The number of yielded lymph nodes was not a significant predictor (*p* = 0.06). The combination of Decipher with PI-RADS score >4 was more strongly associated with PLNM than Decipher score alone (OR = 20.43; 95% CI, 1.78–235.1; *p* = 0.015) or PI-RADS score ≥4 alone (OR = 5.23; 95% CI, 0.67–40.70; *p* = 0.11). Figure 1 and Table 3 depict the ROC curve analysis and AUCs for the associations of interest. There was progressive improvement in the AUC for the detection of PLMN as genomics, and PI-RADS score were combined. Neither AUCs for PI-RADS score ≥4 nor for MRI’s size criteria for identifying PLNM were statistically significant. Decipher score had an AUC of 0.68 (95% CI, 0.58–0.79; *p* = 0.004), but combining PI-RADS score ≥4 with Decipher score led to an AUC of 0.73 (95% CI, 0.63–0.82; *p* < 0.001). The predicted probability for PLNM in patients with Decipher scores >0.8 was the largest at 18%. As Decipher scores increased by increments of 0.2, the predicted probabilities increased linearly from 1.4 to 18%. The overall observed rate of PLNM was (11%). Table 4 demonstrates the predictive probabilities by Decipher score values.

**Table 2.** Multivariate logistic regression model

Predictor	Odds ratio	95% CI	*p*-value
Lymph node yield	1.08	0.98–1.17	0.06
Pre-operative PSA	1.06	1.002–1.11	**0.041**
Decipher score+PI RADS ≥ 4	20.41	2.10–198.74	**0.009**
Biopsy ISUP-GG			
GG 1 (Referent)	-	-	-
GG 2	2.19	0.24–19.75	0.48
GG3	1.56	0.16–15.28	0.70
GG4	2.51	0.24–25.38	0.44
GG5	2.39	0.20–28.13	0.48

CI = confidence interval; ISUP-GG = International Society of Urological Pathology Grade Group; PI-RADS = Prostate Imaging Reporting and Data System;PSA, prostate-specific antigen.

**Table 3.** AUC analysis

Predictor	AUC	95% CI	*p*-value
MRI identification of PLNM	0.60	0.47–0.74	0.09
PI-RADS ≥4	0.60	0.49–0.70	0.14
Decipher score	0.68	0.58–0.79	**0.004**
Decipher score+PI RADS ≥4	0.73	0.63–0.82	**<0.001**

AUC = area under the curve; CI = confidence interval; PI-RADS = Prostate Imaging Reporting and Data System;PLNM, pelvic lymph node metastasis.

**Table 4.** Predicted probabilities of pelvic lymph node metastasis

Predictor	Predicted probability
Decipher score (0.8–1) + PI RADS >4	18%
Decipher score (0.6–0.8) + PI RADS >4	11%
Decipher score (0.4–0.6) + PI RADS >4	6%
Decipher score (0.2–0.4) + PI RADS >4	2.5%
Decipher score (0–0.2) + PI RADS >4	1.4%

PI-RADS, Prostate Imaging Reporting and Data System.

### Subset analysis of PLNM+ patients

**Figure 2.**
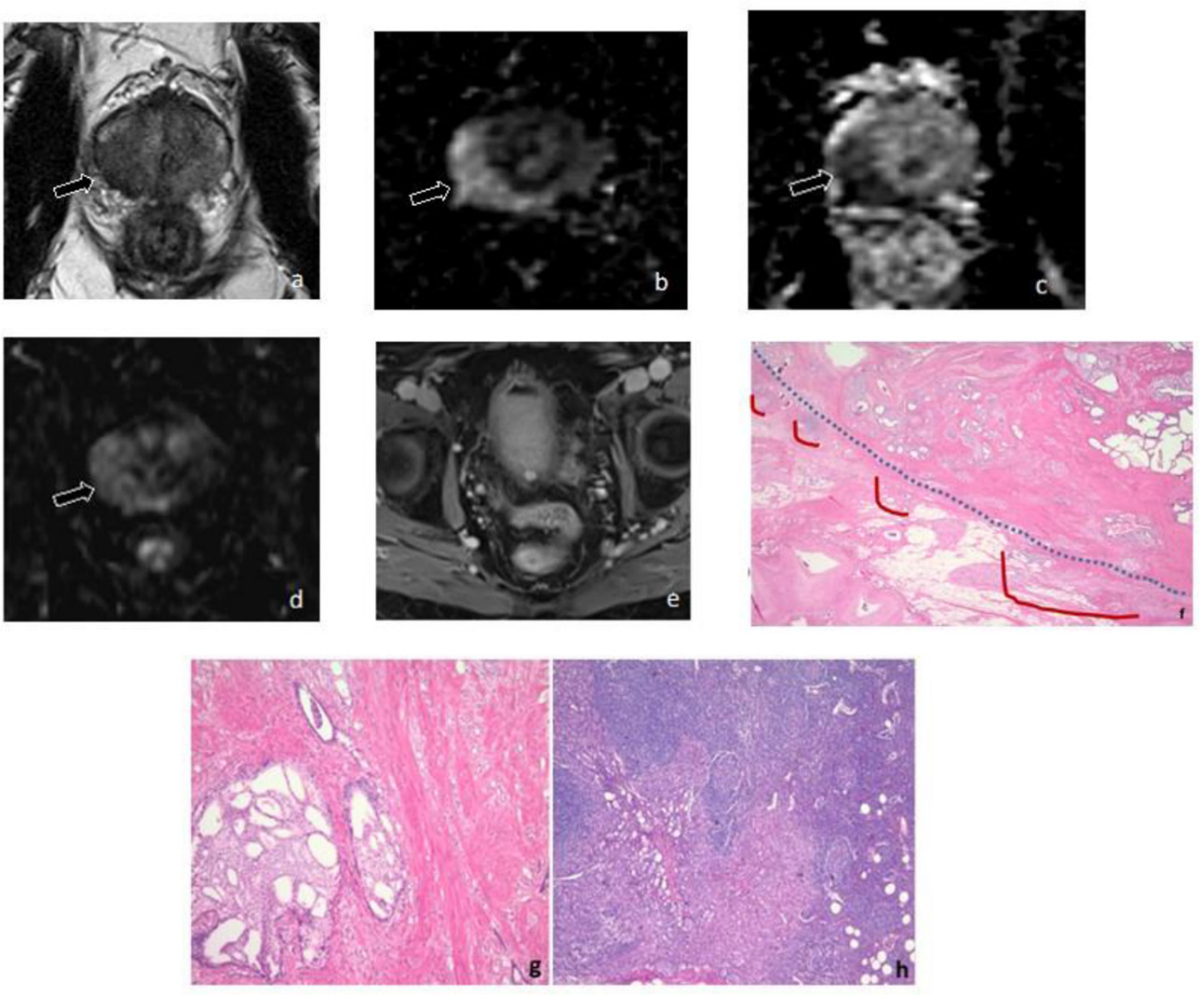
Patient with MRI-invisible PLNM. A 66-year-old man had a prostate-specific antigen level of 2.09 ng/mL and ISUP-GG 2 prostate cancer on biopsy. (A) Axial *T*_2_WI shows diffuse low-signal intensity in the peripheral zone and a focal lesion with ill-defined margins (arrow). The lesion has a broad capsular contact and capsular irregularity, which are indirect signs of EPE (*T*_2_WI score 5). (B) The lesion measures 2.2 cm and has markedly hyperintense signal on DWI (arrow) and (C) demonstrates markedly hypointense signal on the ADC map (arrow) (DWI/ADC score 5). (D) The lesion has early arterial enhancement on DCE *T*_1_WI (arrow) (DCE positive). (E) Axial large field of view post-contrast *T*_1_WI demonstrates no suspicious lymph nodes. (F) Radical prostatectomy demonstrated ISUP-GG 5 prostate cancer with extraprostatic extension, seminal vesical invasion (pT3b), and negative surgical margin. Pelvic lymph node dissection demonstrated metastatic involvement in 2 of 10 lymph nodes. The Decipher score obtained from the index lesion was 6.75 × 10^–1 (high risk). Area of broad capsular contact (blue dashes) with EPE (red solid line). (G) The tumor contains areas of cribriform and intraductal carcinoma. (H) Histologic section oflymph node with metastatic focus of prostatic adenocarcinoma. ADC, apparent diffusion coefficient; DCE, dynamic contrast-enhanced; DWI, diffusion-weighted imaging; EPE, extraprostatic extension; ISUP-GG, International Society of Urological Pathology Grade Group; PLNM, pelvic lymph node metastasis; *T*_1_WI, *T*_1_ weighted imaging; *T*_2_WI, *T*_2_ weighted imaging.

**Figure 3.**
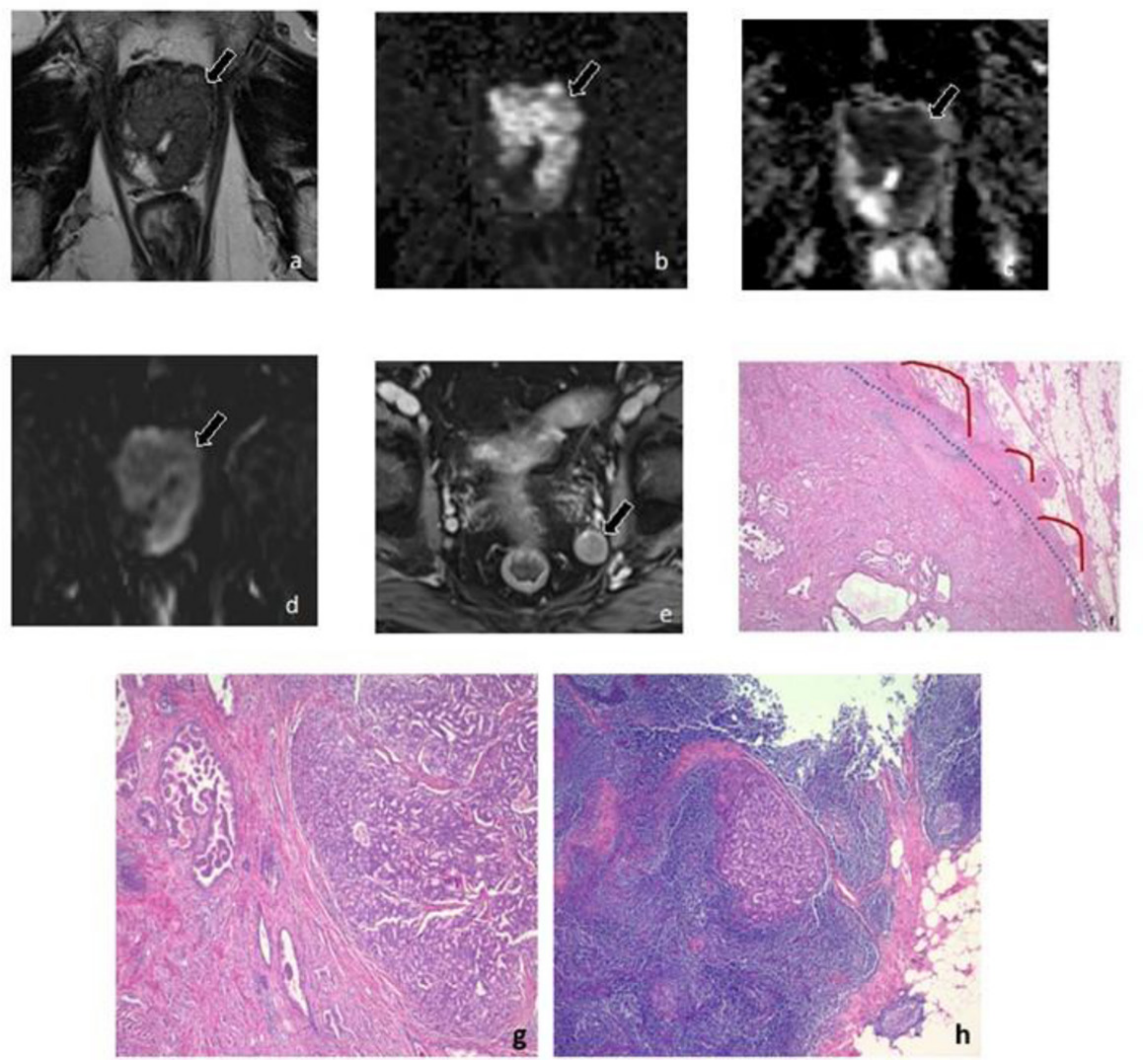
Patient with MRI-visible PLNM. A 70-year-old man had a prostate-specific antigen level of 69.4 ng/mL and ISUP-GG 3 prostate cancer on biopsy. (A) Axial *T*_2_WI shows a hypointense lesion replacing a large portion of the peripheral zone with gross EPE anteriorly (arrow) (*T*_2_WI score 5). (B) The lesion measures 2.2 cm and has markedly hyperintense signal on DWI (arrow) and (C) demonstrates markedly hypointense signal on the ADC map (arrow) (DWI/ADC score 5). (D) The lesion has early arterial enhancement on DCE *T*_1_WI (arrow) (DCE positive). (E) Axial large field of view post-contrast *T*_1_WI demonstrates several suspicious lymph nodes; the largest is in the left internal iliac chain and measures 1.5 × 1.5 cm (arrow). (F) Radical prostatectomy demonstrated ISUP-GG 3 prostate cancer with EPE, microscopic invasion of the bladder neck (pT3a), and positive surgical margin at the apex. Pelvic lymph node dissection demonstrated metastatic involvement in 2 of 16 lymph nodes. The Decipher score obtained from the index lesion was 8.04 × 10^–1 (high risk). Final pathology revealed areas of broad capsular contact (blue dashes) with EPE (red solid line). (G) The tumor contains areas of expansile cribriform glands. (H) Histologic section of lymph node showing metastatic focus of prostatic adenocarcinoma. ADC, apparent diffusion coefficient; DCE, dynamic contrast-enhanced; DWI, diffusion-weighted imaging; EPE, extraprostatic extension; ISUP-GG, International Society of Urological Pathology Grade Group; PLNM, pelvic lymph node metastasis; *T*_1_WI, *T*_1_ weighted imaging; *T*_2_WI, *T*_2_ weighted imaging.

Of the 23 PLNM+ patients, 5 (21.73%) had positive lymph nodes detected at the time of MRI (Supplementary Table 2). Patients with positive lymph nodes on MRI had larger metastatic nodes at final pathology (1.7 cm) than those without (0.3 cm; *p* < 0.001). There was no significant difference in the median Decipher score between patients with positive lymph nodes on MRI (0.87) and those without (0.70; *p* = 0.30). There were no significant differences in the PI-RADS score of MRI visible or invisible lesions (*p* = 0.11). There were no significant differences in the number of positive lymph nodes between MRI visible or invisible lesions (*p* = 0.40). Figures 2 and 3 depict MRI-invisible and MRI-visible PLNM, respectively, and their metastatic diagnosis on lymph node histology.

Supplementary Table 2.

## Discussion

In this study, we found that the combination of elevated Decipher score and PI-RADS score >4 was associated with PLNM, even more so than either modality alone. In addition, the AUC for PLNM detection was significantly higher with this combination than with either individual modality. Finally, predictive probabilities for our outcome of interest demonstrated a progressive rise in PLNM risk when PI-RADS score ≥4 was combined with incremental rises in Decipher risk score ranges (Table 4).

The presence of PLNM at the time of RP is the strongest predictor for biochemical recurrence and cancer-specific mortality in patients with PCa.^20,21^ However, conventional imaging is of limited diagnostic accuracy for the detection of normal-sized PLNM.^22^ In our cohort, only 21.73% of patients (5 of 23) with PLNM were identified pre-operatively on MRI. These results reflect previous findings regarding conventional imaging in this setting, with one meta-analysis of conventional imaging for staging PLNM demonstrating pooled sensitivity and specificity values of 42 and 82%, respectively, for CT and 39 and 82%, respectively, for MRI.^5^ Some MRI enhancements have been made in an attempt to overcome this limitation. For instance, the use of MRI with ultrasmall superparamagnetic iron oxide nanoparticles has shown promise in clinical studies, with a sensitivity for detecting smaller PLNM (short axis, 5–10 mm) of 96.4 *vs* 28.5% with conventional MRI.^23,24^ Implementing the use of these nanoparticles into practice is challenging in terms of cost, feasibility, and practicality, and adoption has remained limited.^24^ The use of PSMA PET for staging high-risk PCa has also shown promise, but the sensitivity of this modality remains low at 0.31–0.42, with a median size cut-off of 6 mm for the detection of PLNM.^6^

This study highlights the potential utility of combining genomic profiling and PI-RADS scores for PLNM assessment. Multivariate logistic regression analysis demonstrated that the combination of an elevated Decipher score and a PI-RADS score ≥4 resulted in an OR of 20.41 (*p* = 0.009) which was higher than other known predictors (*i.e.* PSA, lymph node yield). Although this high ORs is likely in part due to the relatively modest sample size of patients with PLNM (23/202), the statistical significance is noteworthy.

In this study, we found that the median Decipher score was significantly higher in patients with PLMN than in those without. Patients with positive lymph nodes on MRI also demonstrated a higher median Decipher score than those without, but this difference was not statistically significant. From these findings, we infer that Decipher score is correlated with the presence of PLNM but may not necessarily be influenced by the size or volume of PLMN, suggesting the utility of genomic analysis when MRI fails to identify PLNM despite the presence of higher PI-RADS scores.

Multivariate logistic regression analysis demonstrated that PI-RADS scores ≥4 alone was not significantly associated with PLNM (OR = 5.23, *p* > 0.05). Contrary to these results, a previous study found that PI-RADS scores were associated with PLNM on univariate and multivariate analyses (*p* < 0.001).^4^ The differences between our study findings are likely related to differences in the patient populations and the relatively few PLNMs for analysis. The prevalence of ISUP-GG1 PCa at the time of RP in the previous study was 23% (46 of 200) compared to only 1.6% (3 of 179) in our RP cohort. Because PI-RADS scores correlate with PCa GGs, PI-RADS scores should be able to stratify the risk of PLNM in a more diverse population in terms of PCa GG. However, as guidelines support, RP is not the preferred treatment for low-risk patients, and so the utility of these previous results is difficult to extrapolate to contemporary higher-risk cohorts. The combination of Decipher score and PI-RADS score ≥4 is an intriguing option for determining which intermediate/high-risk patients warrant further attention regarding lymph node status. In this study, the AUC for PLNM was 0.73 for the combination of Decipher score and PI-RADS ≥4; although modest, this result is noteworthy when considering the clinicopathological similarities between these higher-risk groups. The predicted probabilities (Table 3) also allow a quantifiable percentage risk for PLNM as Decipher scores increase.

In this study, the median number of nodes retrieved was higher in patients with PLNM than in those without. Multiple factors may explain these findings, including the pre-operative identification of suspicious lymph nodes on MRI or the intraoperative identification of suspicious pathologic nodes. In both situations, this would lead to separate lymph node specimens being sent for evaluation. As shown in studies of other urologic malignancies, sending lymph node specimens separately as opposed to en bloc optimizes the pathologic evaluation and increases the number of lymph nodes described.^25,26^ While our multivariate analysis did not reveal this to be a significant predictor of PLNM, we do feel that with a higher sample size, this would have reached significance. Nevertheless, adding this variable with other pre-operative predictors of PLNM in our analysis did not diminish the association of PI-RADS ≥4 with elevated genomics in predicting PLNM.

Strengths of our study include the unique combination of the highest GG genomic analysis with the PI-RADS scoring system to identify PLNM. These outcomes were evaluated in a contemporary cohort that is higher risk than historical patients and reflects current trends, guidelines recommendations, and changes in practice. Using AUC’s and predictive probability models, we provide meaningful results that can be discussed with patients for counseling and help physicians decide on the need for further advanced imaging (*i.e.* PSMA) or to approach the lymph node dissection in a more extended fashion.

A potential limitation of this study is that the Decipher Scores were performed on the highest grade lesion at RP rather than on biopsy specimens. This raises the initial question of whether a pre-op Decipher score holds predictive accuracy or leads to potential sampling error. Fortunately, spatial heterogeneity does not seem to impact the performance of commonly touted genomic biomarkers.^27^ Furthermore, when comparing biopsy to RP genomic scoring, gene expression profile scores taken from different prostate areas show good correlation between tumors of lower and higher grade.^28^ Thirdly, there is excellent genomic correlation between MRI-targeted biopsies and MRI index lesion on corresponding RP.^29,30^ This last point is important as 97% of highest grade lesions, for which our samples were taken from, are associated with the MRI visible index lesion.^18^ Taken together, these studies support that in patients with MRI-visible high grade tumors, concordance between RP and biopsy Decipher score is highly likely to occur.^28,29^ Finally, due to the retrospective nature of this study, we cannot confirm a standardized lymphadenectomy approach by each surgeon. We appreciate that while lymphadenectomy boundary templates are well known, intraoperative decision-making is a non-measurable but inevitable factor in any surgical event. While a limitation to our study, we tried to circumvent surgical variability by exploring the total number of lymph nodes yielded at final pathology which is a surrogate of the extent of lymph node dissection.

The appropriate treatment of patients with PLNM after RP remains an area of ongoing debate. Although androgen deprivation therapy has been shown to improve survival outcomes in such patients, many urologists elect a conservative approach if PSA remains undetectable.^31^ Improving the pre-operative prediction of PLNM could ultimately ameliorate patient counselling, afford new opportunities for therapeutic planning with enhanced staging adjuncts (*e.g.* PSMA PET), and allow clinicians to tailor their lymph node dissections and treatments. The effect of these changes on survival outcomes is an area of great interest, and prospective research is encouraged.

## Conclusions

The combination of elevated Decipher genomic score (≥0.6) and clinically significant PI-RADS score (≥4) is associated with elevated risks of PLNM at the time of RP in a modern cohort of patients diagnosed with PCa. Such pre-operative tools should be considered when clinicians plan therapeutic avenues for these patients.
