# Supplementary material for: The combination of prostate MRI PI-RADS scoring system and a genomic classifier is associated with pelvic lymph node metastasis at the time of radical prostatectomy
Source: Br J Radiol. 2023 Feb 20;96(1144):20220663. doi: 10.1259/bjr.20220663 (PMC10078867; doi:10.1259/bjr.20220663)
Supplement: Supplementary Table 1. [file bjr.20220663.suppl-01.docx]

**Supplemental Table 1**

Prostate MRI Protocol

| Parameter | Axial T2WI | Sagittal T2WI | Coronal T2WI | Axial DWI^a^ | T1WI DCE MRI^b^ |
| --- | --- | --- | --- | --- | --- |
| Sequence type | TSE | TSE | TSE | SS-EPI | 3D spoiled GRE |
| Field of view (mm) | 180 | 210 | 210 | 200 | 260 |
| Acquisition matrix | 256 × 320 | 269 × 384 | 269 × 384 | 111 × 130 | 166 × 256 |
| Repetition time (ms) | 3730 | 3340 | 3260 | 4700 | 3.29 |
| Echo time (ms) | 121 | 116 | 126 | 86 | 1.26 |
| Flip angle (degrees) | 138 | 120 | 120 | 90 | 15 |
| Slice thickness (mm) | 3 | 3 | 3 | 3 | 3 |

Note—T2WI = T2-weighted imaging, TSE = turbo spin-echo, SS-EPI = single-shoot echo-planar imaging, T1WI = T1-weighted imaging, DCE = dynamic contrast enhanced, GRE =  gradient echo.

^a^b-values: 50, 400, 900, and calculated 1500.

^b^Images obtained after administration of Dotarem 0.2 mL/kg (0.1 mmol/kg) body weight. Temporal resolution: 7-s intervals.
