# Supplementary material for: The combination of prostate MRI PI-RADS scoring system and a genomic classifier is associated with pelvic lymph node metastasis at the time of radical prostatectomy
Source: Br J Radiol. 2023 Feb 20;96(1144):20220663. doi: 10.1259/bjr.20220663 (PMC10078867; doi:10.1259/bjr.20220663)
Supplement: Supplementary Table 2. [file bjr.20220663.suppl-02.docx]

**Supplementary Table 2 – MRI visible vs MRI invisible lymph node invasion**

| Characteristic | MRI visible  (*n* = 5) | MRI invisible  (*n* = 18) | *p* value |
| --- | --- | --- | --- |
| Median diameter of largest lymph node metastasis, cm (IQR) | 1.7 (1.4-2.5) | 0.3 (0.2-0.5) | **< 0.001** |
| PI-RADS scores, n (%) | - | - | 0.11 |
| 1 | 0 (0) | 0 (0) |  |
| 2 | 1 (20) | 0 (0) |  |
| 3 | 0 (0) | 0 (0) |  |
| 4 | 0 (0) | 3 (17) |  |
| 5 | 4 (80) | 15 (83) |  |

IQR= Interquartile Range
